# Divergent Selection Drives Genetic Differentiation in an R2R3-MYB Transcription Factor That Contributes to Incipient Speciation in *Mimulus aurantiacus*

**DOI:** 10.1371/journal.pgen.1003385

**Published:** 2013-03-21

**Authors:** Matthew A. Streisfeld, Wambui N. Young, James M. Sobel

**Affiliations:** Institute of Ecology and Evolution, University of Oregon, Eugene, Oregon, United States of America; Harvard University, United States of America

## Abstract

Identifying the molecular genetic basis of traits contributing to speciation is of crucial importance for understanding the ecological and evolutionary mechanisms that generate biodiversity. Despite several examples describing putative “speciation genes,” it is often uncertain to what extent these genetic changes have contributed to gene flow reductions in nature. Therefore, considerable interest lies in characterizing the molecular basis of traits that actively confer reproductive isolation during the early stages of speciation, as these loci can be attributed directly to the process of divergence. In Southern California, two ecotypes of *Mimulus aurantiacus* are parapatric and differ primarily in flower color, with an anthocyanic, red-flowered morph in the west and an anthocyanin-lacking, yellow-flowered morph in the east. Evidence suggests that the genetic changes responsible for this shift in flower color have been essential for divergence and have become fixed in natural populations of each ecotype due to almost complete differences in pollinator preference. In this study, we demonstrate that a *cis*-regulatory mutation in an R2R3-MYB transcription factor results in differential regulation of enzymes in the anthocyanin biosynthetic pathway and is the major contributor to differences in floral pigmentation. In addition, molecular population genetic data show that, despite gene flow at neutral loci, divergent selection has driven the fixation of alternate alleles at this gene between ecotypes. Therefore, by identifying the genetic basis underlying ecologically based divergent selection in flower color between these ecotypes, we have revealed the ecological and functional mechanisms involved in the evolution of pre-mating isolation at the early stages of incipient speciation.

## Author Summary

A fundamental goal of evolutionary biology is to identify the genetic changes and evolutionary mechanisms involved in speciation. Knowledge of the genes allows us to address important unresolved questions about the genetics of speciation, such as what role does ecologically based natural selection play during the process of divergence? We characterize the evolutionary processes and genetic changes that influence the early stages of speciation between divergent populations of the wildflower *Mimulus aurantiacus*. Populations with different flower colors attract different animal pollinators, resulting in a prominent reproductive barrier that reduces gene exchange between populations. We provide multiple lines of corroborating evidence to verify the functional role of the major gene responsible for this flower color shift, including genetic mapping, gene expression analyses, and gene knockdown approaches. We then use molecular population genetic techniques to show a remarkably tight association between genetic variants in this gene and the geographic shift in flower color, which reflects a history of divergent natural selection on flower color between these populations. These results provide a striking demonstration of how adaptation to different environments can result in the early stages of divergence and the evolution of reproductive isolation between populations.

## Introduction

Revealing the specific genes and mutations that underlie reproductive isolation provides a window into the evolutionary and molecular mechanisms that drive speciation. Characterization of these genes allows us to explore several long-standing questions about the genetics of speciation, such as how many genetic changes underlie individual isolating traits and what are their relative effect sizes [1]? What role does genetic architecture play in speciation [2]? And, have the genetic changes been fixed due to natural selection [3], [4]?

Ecologically-based divergent selection appears to be a pervasive feature of speciation that leads to the evolution of reproductive isolation as a by-product of adaptation to different environments [5]–[7]. However, despite much evidence for natural selection's role in the evolution of reproductive isolation, the genetic changes that underlie these adaptive traits have been characterized in only a few cases [8]. On the other hand, signatures of positive selection have been detected at some of the genes contributing to reproductive isolation, but in these examples, the ecological mechanisms underlying this selection are rarely known [9]–[11]. Therefore, to understand how divergent selection has influenced the speciation process, it is necessary to integrate ecological studies of traits involved in reproductive isolation with molecular and population genetic techniques that can identify the evolutionary forces and genetic changes controlling variation in these traits. By connecting these approaches, it becomes possible to identify the functional and ecological mechanisms that guide the evolution of new species [8].

Most examples that describe the genetic basis of speciation involve retrospective analyses between completely isolated species [12]. However, because the traits contributing to reproductive isolation continue to accumulate after speciation is complete, it is difficult to determine which of these components of isolation were the drivers of divergence and which arose following speciation [13]. In addition, even though the genetic changes controlling intrinsic postzygotic barriers have been characterized among several species [14], [15], in many cases, these taxa are allopatric, which suggests that these barriers do not actively contribute to gene flow reductions in nature [16]. By contrast, regions of natural hybridization that develop between partially isolated incipient species are recognized as ‘natural laboratories’ because the efficacy of natural selection to maintain barriers is tested constantly in the face of ongoing gene flow [17], [18]. Moreover, because gene exchange persists, hybrid zones have the potential to reveal the genetic changes influencing divergence before they become confounded with other species differences. Nevertheless, despite the rich theoretical and empirical history of clines and hybrid zones [19]–[21], little is known about the form and intensity of selection acting on the specific genes affecting reproductive isolation between divergent taxa.

Relative to other forms of reproductive isolation, adaptations that reduce the frequency of mating among neighboring populations (i.e. pre-mating barriers) can have disproportionate effects on reducing gene flow, particularly if they arise during the early stages of divergence [5], [22]. However, comparatively few examples exist where the molecular genetic basis of a pre-mating isolating barrier has been characterized [12], [14]. Pollinator isolation is widely regarded as a common mechanism of adaptation and speciation in plants [23], [24], and flower color has been implicated as one of the primary traits involved [25]. However, of the most highly regarded examples describing the role of flower color in driving pollinator isolation, either the precise genetic changes have not yet been identified [26], the flower color shift occurred as a reinforcing mechanism to complete the speciation process [27], or it occurred after speciation was complete [28], [29]. In addition, while the genetic basis of flower color differences has been characterized in other cases [30], [31], there is no evidence from these studies that pollinator-mediated selection drove flower color change. Therefore, considerable interest lies in identifying the molecular basis of traits such as flower color that actively confer pre-mating isolation at the early stages of divergence.

**Figure 1:**
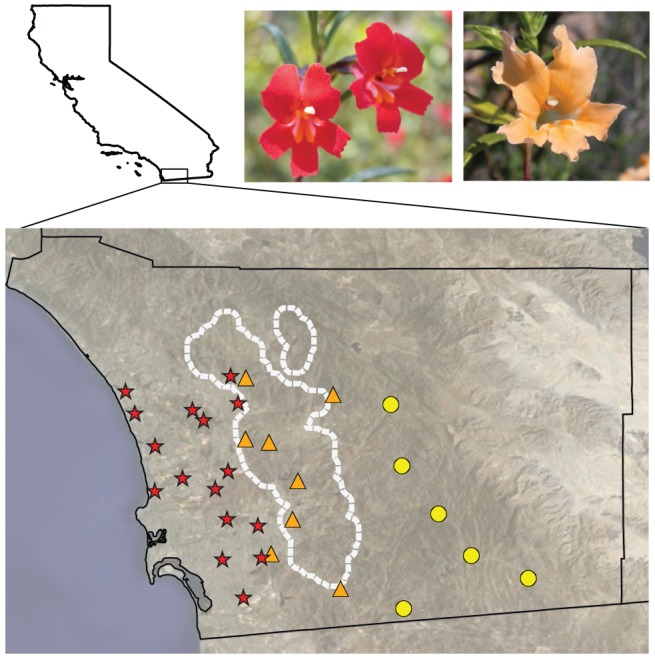
Flower color varies geographically in *M. aurantiacus*. Representative pictures of red and yellow ecotype flowers in San Diego County, California. Locations of the red (red stars) and yellow ecotype populations (yellow circles) and their natural hybrids (orange triangles) sampled in this study. The position of the hybrid zone was determined previously [33], and is outlined in white.

In *Mimulus aurantiacus* (Phrymaceae), there is strong evidence that geographic variation in flower color contributes to reproductive isolation between two ecotypes distributed along an east-west gradient in Southern California (Figure 1). In this classic example of incipient speciation [24], [25], the ecotypes remain highly isolated due to differences in pollinator behavior, with hummingbirds showing greater than 95% preference for a red-flowered ecotype that occurs in the west and hawkmoths showing nearly complete preference for a yellow-flowered ecotype in the east [25], [32]. The incomplete nature of this barrier allows a hybrid zone to occur where the two ranges overlap, resulting in gene flow between ecotypes at neutral loci [33]. Moreover, intrinsic postzygotic barriers to gene flow are weak [34], suggesting that isolation occurs predominantly from divergent selection associated with differences in pollinator preference. Therefore, the genetic changes responsible for the shift in flower color appear to have been essential to the early stages of divergence in this system.

**Figure 2:**
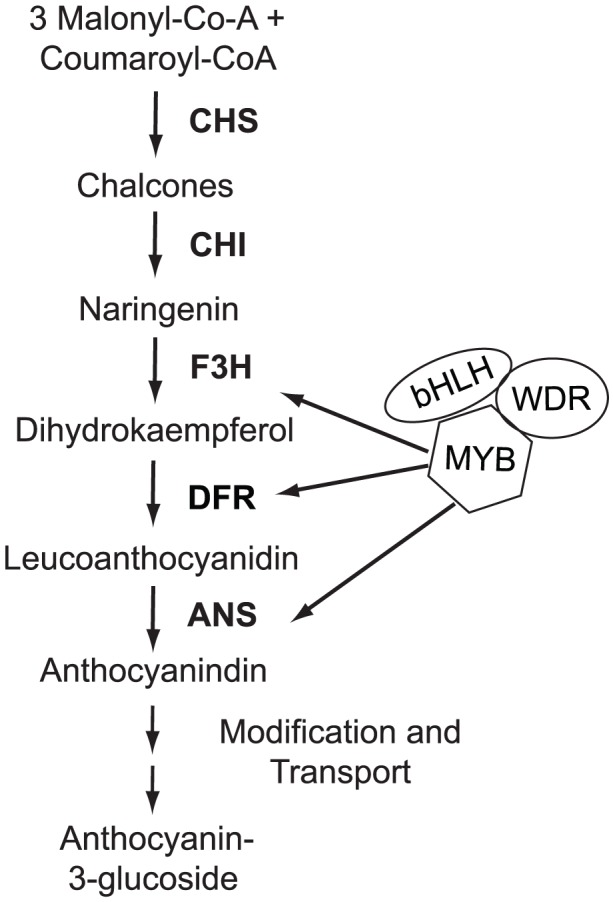
Flower color variation in *M. aurantiacus* is caused by differences in anthocyanin pigmentation. A schematic of the anthocyanin biosynthetic pathway and its regulation. Abbreviations: CHS, chalcone synthase; CHI, chalcone isomerase; F3H, flavonol 3-hydroxylase; DFR, dihydroflavonol 4-reductase; ANS, anthocyaninidin synthase. R2R3-MYB (MYB), basic helix-loop-helix (bHLH), and WD-40 repeat (WDR) transcription factors regulate enzymatic expression.

The red and yellow ecotypes both produce yellow, carotenoid pigments in their flowers, but only the red ecotype produces anthocyanins [35]. Thus, the major difference in flower color between the ecotypes is caused by the presence versus absence of anthocyanins. Anthocyanin floral pigments are synthesized by a well-characterized pathway that is highly conserved across angiosperms [36]. Anthocyanin biosynthesis requires the function of at least six enzymes, many of which are coordinately regulated by three interacting transcription factor proteins (Figure 2) [37]. Therefore, to generate the changes in anthocyanin pigment intensity that are responsible for red versus yellow flowers between the ecotypes, flux through this pathway must be altered. In principle, such alteration may be caused in any of four ways: (i) by *cis*-regulatory changes to enzyme-coding genes that directly affect enzymatic expression; (ii) by coding-sequence mutations in enzyme-coding genes; (iii) by *cis*-regulatory changes to anthocyanin transcription factors; or (iv) by coding-sequence mutations to anthocyanin transcription factors [38]. Despite these possibilities, the repeated evolution of flower color transitions among angiosperm species reveals a striking pattern of genetic convergence, with all known cases of shifts in floral anthocyanin intensity resulting from mutations in one class of pigment-regulating transcription factor [38].

We have shown previously that flower color variation in *M. aurantiacus* is a quantitative character resulting from mutations in at least two interacting loci that affect the expression levels of three essential pathway enzymes [35]. Allelic differences at one of these loci explained 45% of the phenotypic variation in floral anthocyanin content and contained the gene encoding the anthocyanin pathway enzyme, dihydroflavonol 4-reductase (*MaDfr*). Despite significantly higher *MaDfr* expression in red compared to yellow flowers, functional analyses suggested that an unknown genetic factor was responsible for these changes in gene expression and ecotypic differences in floral anthocyanin pigmentation [35]. Because this locus affects expression of multiple pathway enzymes including *MaDfr*, it was hypothesized to encode a transcription factor protein. Moreover, because flower color variation mapped to a genomic region of major effect containing *MaDfr*, this gene was presumed to be linked to *MaDfr*. However, the identity of this factor and the type of mutation (i.e. *cis*-regulatory or coding) affecting flower color differences were not determined.

With ample ecological motivation in place [32], [33], we take advantage of the resources available in the anthocyanin pathway and combine classic genetic techniques, gene expression analyses, and gene knockdown approaches to characterize the molecular genetic basis of incipient species differences in flower color. We then use molecular population genetic data to demonstrate how natural selection has driven the fixation of alleles at this gene between two ecotypes at the early stages of divergence. Our results reveal the power of combining ecological and molecular investigations to the study of speciation genetics, as we not only identify a major gene associated with the evolution of reproductive isolation, but we also demonstrate how natural selection can drive ecological divergence integral to the speciation process.

## Results

### Examination of congeneric *M. guttatus* reveals potential candidate regulators in the R2R3-MYB family of transcription factors

**Figure 3:**
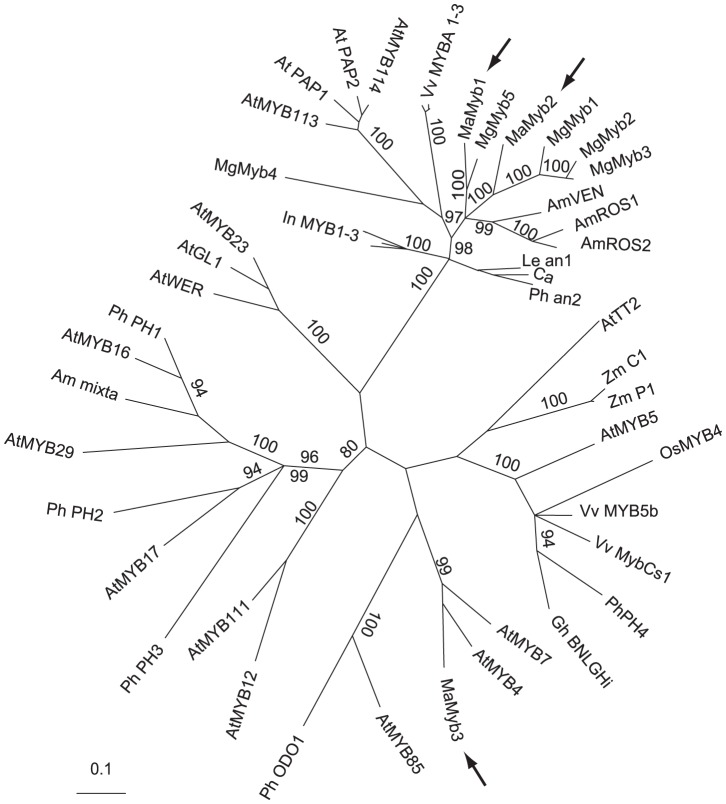
*MaMyb2* is homologous with other known anthocyanin-regulating R2R3-MYB genes. An unrooted, majority rule consensus gene tree of the R2R3 DNA binding domain among a subset of known R2R3-MYB genes is shown. With the exception of *MaMyb2* and *MaMyb3* (both described in this study) and *MgMyb1-5*, all other sequences and details of phylogenetic reconstruction are the same as in [35]. *MgMyb1-5* sequences were obtained from [31]. Arrows denote the locations of the three floral-expressed genes identified in *M. aurantiacus*. Numbers along branches indicate posterior probabilities (×100) of branch support from the Bayesian analysis. Only posterior probabilities ≥80 are reported.

We used the genome sequence available from the closely related *M. guttatus* to search for putative pigment regulators linked to the single copy *Dfr* gene (*MgDfr*). No anthocyanin pigmentation candidates were located on the sequence scaffold containing *MgDfr*. However, three R2R3-MYB transcription factors (*MgMyb1-3*) homologous to known anthocyanin regulators from model species [39], were distantly linked to *MgDfr*. By screening *M. aurantiacus* floral complementary DNA (cDNA), we identified three R2R3-MYB-related genes expressed in *M. aurantiacus* floral tissue (*MaMyb1-MaMyb3*). The sequence of *MaMyb1* was described previously [35]. To infer homology of these genes to other known anthocyanin regulators, we constructed gene trees, as described previously [35]. *MaMyb1* and *MaMyb2* grouped in a highly supported clade containing known anthocyanin regulators from subgroup six of the R2R3-MYB family [39] (Figure 3). By contrast, *MaMyb3* did not group with the anthocyanin regulators, as it formed a clade with sequences from *Arabidopsis* subgroup four [41].

### Genetic mapping identifies *MaMyb2* as a prime candidate controlling the flower color shift

**Figure 4:**
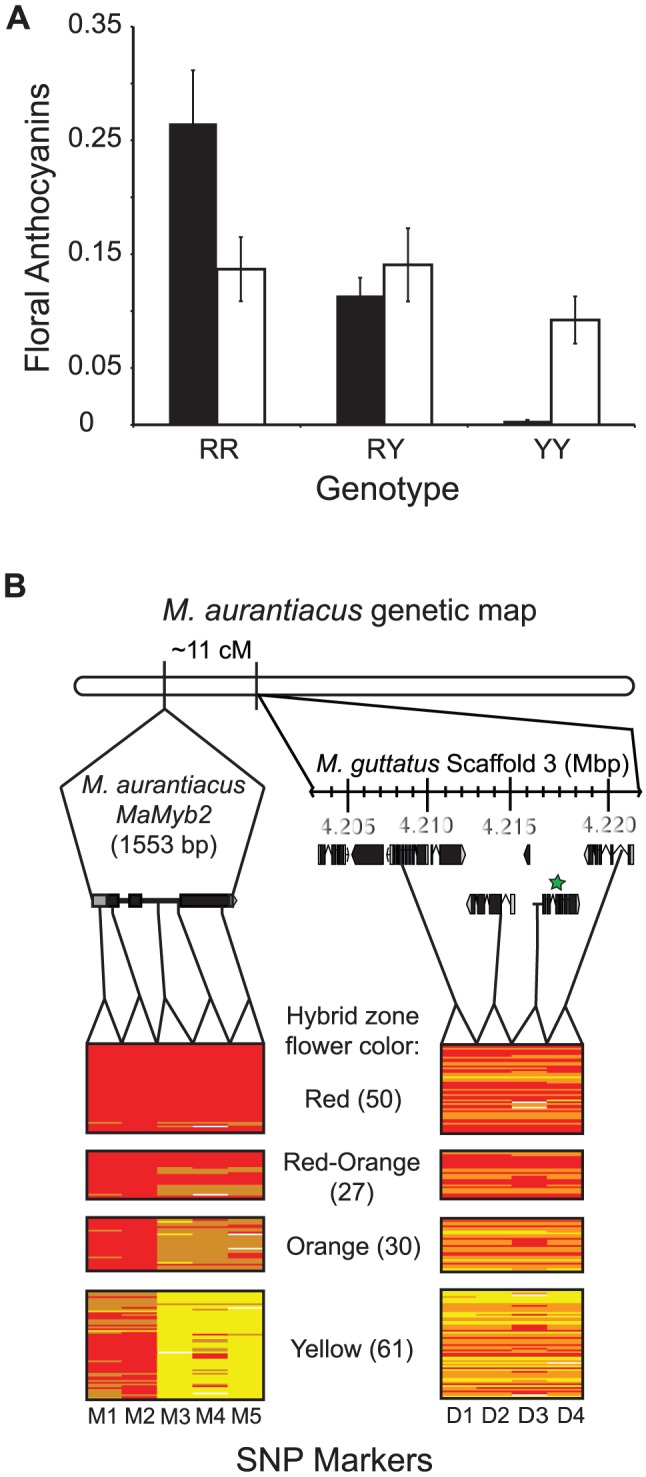
Genetic mapping implicates *MaMyb2* in the flower color change. *(A)* Among 79 recombinant F_2_ hybrids, genotype at *MaMyb2* (black bars) predicts floral anthocyanin content (F_2,76_ = 20.66; *P*<0.0001), but genotype at *MaDfr* (white bars) does not (F_2,76_ = 0.53; *P* = 0.59). Error bars indicate one standard error. *(B)* From 168 naturally-occurring hybrid plants, the relationship between genotype (*RR* = red bars; *RY* = orange bars; *YY* = yellow bars; missing data = white bars) and flower color is shown for SNP markers in *MaMyb2* (M1–M5) and surrounding the linked *MaDfr* (D1–D4). The genetic map in *M. aurantiacus* is shown, demonstrating that *MaMyb2* and *MaDfr* are separated by 11 cM. Insets depict the physical positions and gene structures (boxes = exons; lines = introns) of the M1–M5 and D1–D4 markers on different scales. Relative positions of the markers were identified either by sequencing *MaMyb2* in *M. aurantiacus* (1553 bp) or from scanning the *M. guttatus* genome assembly (v. 1.1) scaffold 3 that contains *MgDfr* (region spanning ∼15 kb). All markers were linked to each other and to *MaDfr* in *M. aurantiacus*. The green star indicates the *MaDfr* gene. Functional annotations for the genes containing markers D1–D4 are described in Table S3.

While mutations in at least two loci are involved in flower color differences between the ecotypes [35], we focus here only on the genetic basis of the locus explaining the greatest variation in flower color. Because our previous data suggested that this major regulator should be linked to *MaDfr* in *M. aurantiacus*, we established linkage relationships among *MaMyb1*, *MaMyb2*, *MaMyb3*, and *MaDfr* in 359 F_2_ hybrids derived from a cross between the ecotypes. Among the three floral-expressed R2R3-MYB genes, *MaMyb1* and *MaMyb3* were unlinked to *MaDfr*, but *MaMyb2* was located approximately 11 cM from *MaDfr* (Table S1). Genotypes at these and all subsequent markers used in this study (see below) were classified as *RR*, *RY*, or *YY* depending on whether alleles were derived from the red-ecotype (*R*) or the yellow-ecotype (*Y*) parents in the cross. Consistent with these patterns of linkage, genotype at *MaMyb2* was significantly associated with total anthocyanin content in the flowers of these same F_2_ plants, with *MaMyb2* explaining approximately 50% of the phenotypic variation in flower color (Table S2; F_(2,357)_ = 176.33; *P*<0.0001). Among the 79 observed recombinants between *MaMyb2* and *MaDfr* in this F_2_ cross, *MaMyb2* genotype significantly predicted differences in floral anthocyanins, but *MaDfr* genotype did not (Figure 4A), suggesting that the major flower color locus resides in the genomic region containing *MaMyb2*. While additive or epistatic effects of mutations in *MaDfr* may still account for a small percent of the variation in flower color between the ecotypes, linkage between the genes precluded a formal analysis of these potential minor effects in this study.

### Genetic variation in *MaMyb2* is associated with flower color in natural hybrid zones

To more precisely characterize the major genetic locus contributing to flower color evolution, we harnessed the power of natural hybrid zones for genotype-phenotype association studies. Ample opportunity for recombination in hybrid zones breaks down linkage disequilibrium, such that positive associations likely represent tight linkage with functional mutations. We genotyped five single nucleotide polymorphisms (SNPs) spanning a distance of 1383 bp within *MaMyb2* (M1–M5) and four additional SNPs in genes surrounding *MaDfr* (D1–D4) (Table S3) from 168 plants from eight hybrid populations where flower color variation segregated (Figure 1; Table S4). Genetic variation at markers M3–M5 had a remarkably tight association with flower color in the hybrid zone (range *P* = 10^−43^ to *P* = 10^−55^) (Figure 4B; Table S5), with nearly perfect agreement between the phenotype and that predicted by genotype. Genotypes at D1–D4 and M1–M2 were also significantly associated with flower color (range *P* = 10^−5^ to *P* = 10^−9^), but these associations were an order of magnitude weaker than those for M3–M5. Variation in the strength of these associations among markers likely reflects the quantitative nature of this trait and the impact of one or more factors that influence the maintenance of linkage disequilibrium between loci, including the distance to the causal mutation, the strength, timing, and evolutionary history of natural selection, and the local recombination rate [42]. Regardless, these data provide robust support for the hypothesis that genetic variation in *MaMyb2* contributes to ecotype differences in flower color.

### Gene expression is associated with genotype at *MaMyb2*

**Figure 5:**
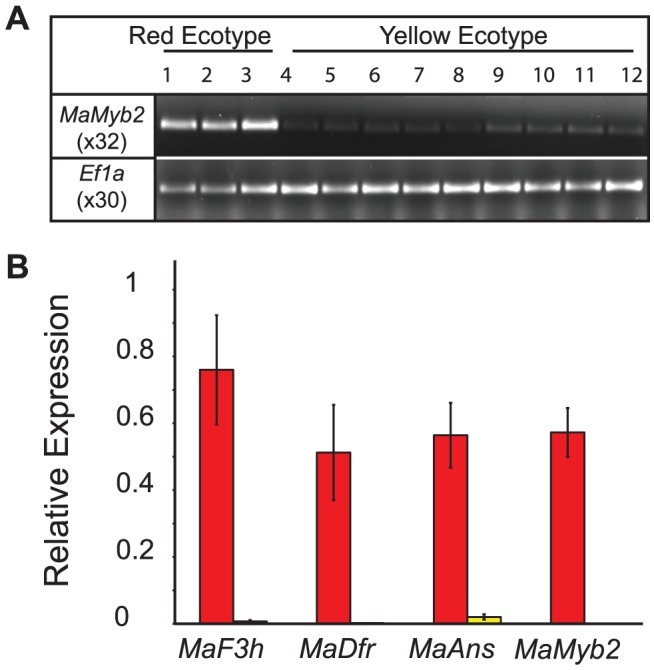
Gene expression is associated with genotype at *MaMyb2*. *(A) MaMyb2* floral expression is qualitatively associated with elevated floral anthocyanin content in red and yellow flowers collected from natural populations. PCR cycle numbers are listed below each gene. *Ef1a* is a constitutively expressed control. *(B)* In 18 F_3_ hybrids, gene expression segregates with genotype at *MaMyb2* and is significantly higher in flowers that are *RR* (red bars) compared to *YY* (yellow bars) (F*_MaF3h_*_ (1, 16)_ = 16.76, *P* = 0.0008; F*_MaDfr_*_(1, 16)_ = 10.12, *P* = 0.006; F*_MaAns_*_ (1, 16)_ = 24.82, *P*<0.0001; F*_MaMyb2_*_ (1, 16)_ = 48.00, *P*<0.0001). Error bars indicate one standard error.

The genetic association between flower color and *MaMyb2* genotype also corresponds to strong differences in gene expression. In flowers collected from natural populations, *MaMyb2* expression was consistently associated with elevated floral anthocyanin content (Figure 5A). Moreover, among F_3_ hybrids, genotype at *MaMyb2* segregated significantly with its own expression and the expression levels of three anthocyanin pathway enzymes essential for pigment production [35] (Figure 5B).

### *MaMyb2* is necessary for floral anthocyanin pigmentation

**Figure 6:**
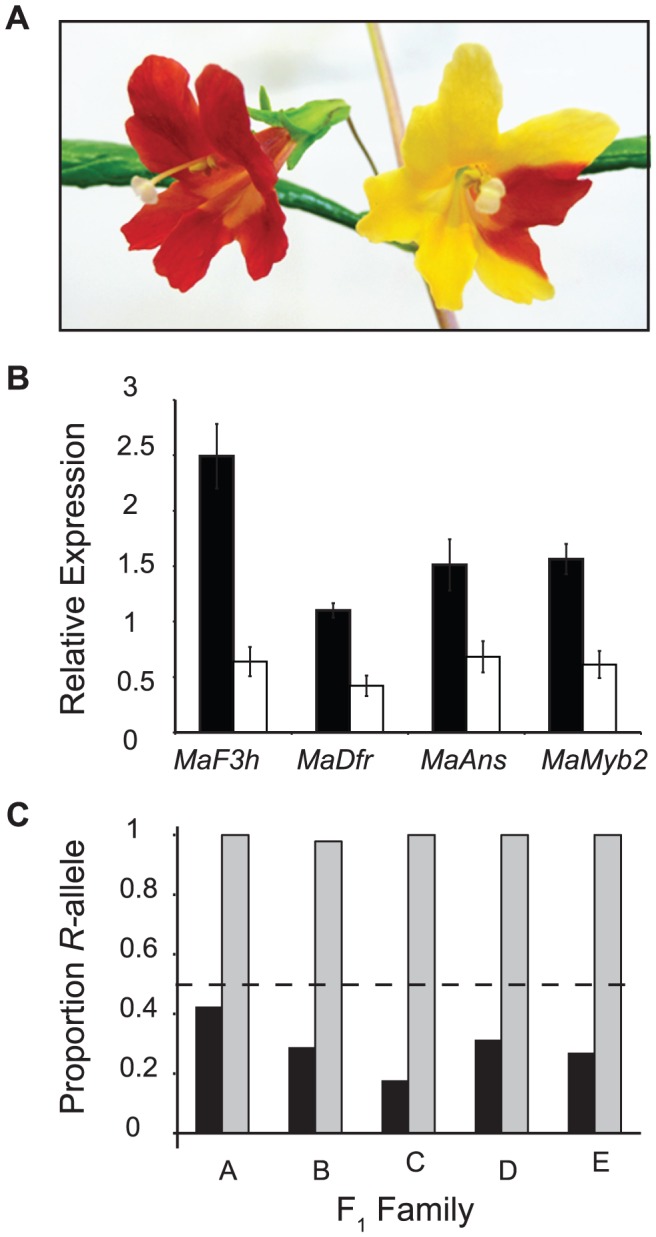
A *cis*-regulatory mutation in *MaMyb2* alters anthocyanin enzyme expression and flower color. *(A)* Wild-type (left) and *MaMyb2*-silenced (right) flowers from VIGS experiments. See Figure S1 for additional pictures demonstrating the range of silencing. *(B)* Compared to pTRV2 negative controls (black bars), *MaMyb2* VIGS silencing (white bars) leads to significantly fewer floral transcripts of *MaMyb2* and the enzymes it regulates (F*_MaF3h_*_ (1, 10)_ = 46.07, *P*<0.0001; F*_MaDfr_*_(1, 10)_ = 23.38, *P* = 0.0007; F*_MaAns_*_(1, 10)_ = 10.41, *P* = 0.009; F*_MaMyb2_*_(1, 10)_ = 22.72, *P* = 0.0008). Error bars are one standard error. *(C)* Among five independent F_1_ crosses, the *R*-allele at *MaMyb2* is represented in floral cDNA at significantly higher levels than the *Y*-allele [cDNA (grey bars) compared to gDNA (black bars), Fisher's exact test: *P*<10^−9^ in all families; cDNA compared to equal contribution of both alleles (dashed line), Fisher's exact test: *P*<10^−4^ in all families]. This indicates that a *cis*-regulatory mutation in *MaMyb2* accounts for the signficant differences in *MaMyb2* floral expression.

The concurrence of genetic mapping and differential regulation of anthocyanin pathway enzymes firmly point to altered expression of *MaMyb2* as the major genetic change responsible for adaptive flower color evolution. Therefore, as a first step to establish that genetic variation in *MaMyb2* was responsible for the differences in flower color between the ecotypes, we post-transcriptionally silenced *MaMyb2* in plants of the red ecotype using Virus-Induced Gene Silencing (VIGS) [43]. As expected for a regulator of the anthocyanin pathway, petal cells containing the *MaMyb2* silencing construct produced no anthocyanins, which caused the usually hidden yellow carotenoid pigment to become visible in the red ecotype flowers (Figure 6A). Furthermore, significantly reduced *MaMyb2* expression among silenced flowers relative to negative controls led to concomitant reductions in the expression levels of *MaF3h, MaDfr*, and *MaAns*, suggesting that these genes were regulated by *MaMyb2* (Figure 6B).

While it is possible that the recovered VIGS phenotype is caused by the unintended silencing of additional R2R3-MYB genes by our VIGS construct, this situation is unlikely. In particular, we engineered the silencing construct to contain little sequence similarity between *MaMyb2* and the other two R2R3-MYB genes known to be expressed in flowers (Figure S1). Furthermore, gene expression of *MaMyb2*, *MaF3h, MaDfr*, and *MaAns* co-segregated with genotype at *MaMyb2* in F_3_ lines (Figure 5B), suggesting that variation in *MaMyb2* expression (or something linked to it) regulates these enzymes. However, because *MaMyb1* and *MaMyb3* are not linked to *MaMyb2*, they cannot account directly for gene expression differences explained by *MaMyb2* genotype. Therefore, these results indicate that *MaMyb2* is necessary for the proper synthesis of red pigmentation in flowers via its effect on the expression of the anthocyanin enzymes.

### A *cis*-regulatory mutation in *MaMyb2* controls floral color

Despite confirmation that *MaMyb2* is an integral component of the floral anthocyanin regulatory network in *M. aurantiacus*, it is necessary to examine whether *MaMyb2* is a downstream target of a linked transcription factor that is directly responsible for flower color change. To determine whether differences in *MaMyb2* floral expression could be attributed to *cis*- or *trans*-acting mutations, we examined variation in allele-specific expression among F_1_ heterozygotes. A significant allelic imbalance would indicate *cis*-regulation, whereas equal expression of both alleles would demonstrate a mutation acting in *trans* [44]. In five independent F_1_ hybrids, both *R* and *Y* alleles were amplified in genomic DNA samples. By contrast, the *R* allele was expressed in floral cDNA samples at significantly higher levels than the *Y* allele (Figure 6C), suggesting that differences in *MaMyb2* expression between the ecotypes are due to a *cis*-acting mutation in *MaMyb2* and not a linked *trans*-factor.

While these data support the hypothesis that flower color differences are primarily attributed to a *cis*-regulatory mutation in *MaMyb2*, full-length sequencing of the *MaMyb2* coding region from red and yellow ecotypes revealed nine non-synonymous and two in-frame insertion-deletion substitutions (Figure S2). Any or all of these coding mutations could have altered the function of the *MaMyb2* protein prior to the *cis*-regulatory change that affects gene expression. To address this possibility, we sequenced the *MaMyb2* coding region from *Mimulus aridus* and *M. clevelandii* (sensu [45]), two closely-related, yellow-flowered taxa that co-occur with the red and yellow ecotypes in San Diego County, California. These taxa are members of the section Diplacus, a clade of several closely related, perennial shrubs distributed across California. Even though the phylogenetic relationships among Diplacus taxa are unclear at this time [46], the flowers of *M. aridus* and *M. clevelandii* lack anthocyanins. Thus, if any of the variable sites in the red ecotype are shared with these yellow-flowered taxa, this suggests it is unlikely for these sites to be functionally related to differences in floral anthocyanin production between the ecotypes.

Only one of the coding changes occurred in the conserved R2R3 DNA binding domain that is responsible for contacting the promoter of its target genes [41]. At this site (site 22; Figure S2, which is also SNP marker M2, see above), the *M. aridus* and *M. clevelandii* sequences were the same as the red ecotype sequences. In addition, 41 yellow-flowered plants from the natural hybrid zone were homozygous for the *R* allele at this SNP (Table S3), further suggesting that it is not responsible for differences in flower color. Moreover, with the exception of only one of the remaining coding changes (position 203), none of the sites were unique to the red ecotype (Figure S2), suggesting that they are unlikely to be functionally associated with floral anthocyanin production. While we cannot directly rule out the coding change at position 203 as being important for *MaMyb2* function, it is worth noting that it involves a conservative change between two polar, uncharged amino acids (threonine and asparagine). Therefore, the combination of allele-specific expression and comparative sequencing data suggests that a *cis*-regulatory mutation in *MaMyb2* is directly responsible for the major genetic change in floral anthocyanin pigmentation between the red and yellow ecotypes.

### Elevated allelic differentiation across the flower color locus

Locus-specific patterns of elevated genetic differentiation between populations are indicative of positive selection [42]. We genotyped the nine SNPs described above (M1–M5 and D1–D4) in an additional 374 individuals from 22 populations outside of the hybrid zone (Figure 1; Table S4). Alleles at all five markers across *MaMyb2* (M1–M5) were highly differentiated between ecotypes (F_ST_>0.808) (Table 1). In particular, SNP M5 was differentially fixed in western and eastern populations for *R* and *Y* alleles, respectively. We also found very high allelic differentiation at D1–D4 (F_ST_ range: 0.658–0.711) even though genetic variation in *MaDfr* did not appear to be responsible for ecotypic differences in flower color. By contrast, the mean between-ecotype F_ST_ calculated previously from 100 polymorphic AFLP loci at many of these same populations was an order of magnitude lower (F_ST_ = 0.081), with no one locus having an F_ST_ greater than 0.39 [33].

**Table 1:** *R* allele frequencies and F_ST_ calculated between each ecotype from 374 individuals from 22 populations outside of the hybrid zone.

Marker	Frequency *R* allele in red-ecotype populations	Frequency *R* allele in yellow-ecotype populations	Between-ecotype F_ST_
M1	0.996	0.158	0.891
M2	0.998	0.286	0.808
M3	0.994	0.005	0.989
M4	1	0.029	0.981
M5	1	0	1
D1	0.806	0.033	0.711
D2	0.788	0.037	0.685
D3	0.779	0.051	0.658
D4	0.764	0.033	0.658
AFLP*	N/A**	N/A**	0.081

* Calculated previously [33] from 100 AFLP markers from five red ecotype and five yellow ecotype populations.

** Not applicable based on the dominance of AFLP markers.

### Analysis of cline shape reveals signatures of divergent selection

To more precisely evaluate the spatial patterns of genetic variation across both ecotypes and their natural hybrid zone, we used maximum likelihood to estimate six parameters that together defined the shape and geographic position of the allele frequency clines from each of the nine markers [47], [48]. While clines are influenced by a number of factors including drift, selection, and gene flow [49], comparisons of cline shape and position across loci have the potential to distinguish among these processes. For example, clines at neutral loci can be due to recent contact among previously isolated populations, but introgression following contact will tend to widen and eventually flatten clines [20]. On the other hand, steep clines at particular loci can be maintained in the face of gene flow due to selection.

**Figure 7:**
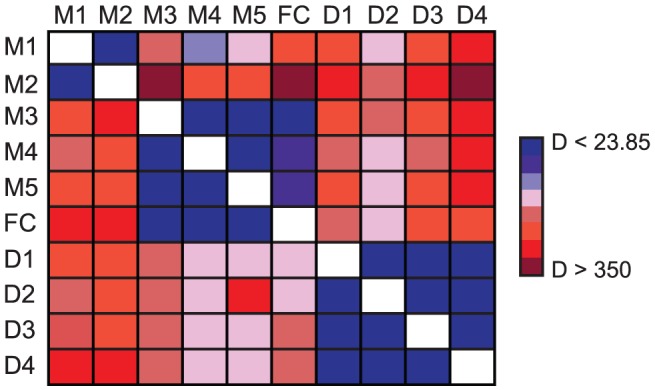
Significant differences in cline shape among markers. Pairwise likelihood ratio tests (LRT) among all SNPs (M1–M5; D1–D4) and flower color (FC) to test for differences in cline shape. For each comparison, the data source listed in each column was constrained to the set of parameters estimated from each row and the log-likelihood of the constrained model was re-estimated. Each cell in the matrix is color-coded according to the magnitude of *D* (two times the difference in log-likelihood values between the freely estimated and constrained model). Each color increases *D* in 50 unit increments. Estimates of *D* less than the critical value for Bonferroni-corrected statistical significance and six degrees of freedom (*D*<23.85) are in blue and denote those comparisons for which cline shape parameters are not significantly different between markers.

**Figure 8:**
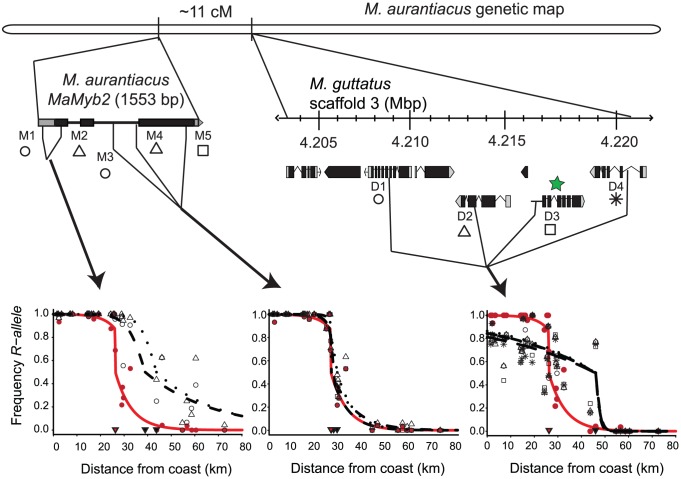
Spatial variation in selection among SNPs surrounding the flower color locus. In all plots, the red curve denotes the cline in flower color estimated from phenotypic data, and black curves denote the allele frequency clines for SNPs. Genetic markers are grouped according to results of pairwise likelihood ratio tests (Figure 7), with each panel combining groups of markers that are not significantly different from each other (left: M1 and M2; center: M3–M5; right: D1–D4). The frequency of red flowers in each population is plotted as red circles in each panel and the frequency of the *R* allele at each SNP in each population is plotted as different black symbols (as indicated below each marker name). Filled triangles indicate the cline center for each SNP (black) and flower color (red) as estimated using maximum likelihood (see Table S6 for parameter estimates). Details of marker positions and maps are the same as in Figure 4. Functional annotations for the genes containing markers D1–D4 are described in Table S3.

We observed characteristic sigmoidal cline shapes for all nine SNPs. However, little or no clinal variation was detected in a previous study investigating geographic variation of allele frequencies at randomly selected nuclear and chloroplast DNA markers from a similar set of populations that spanned the geographic range of both ecotypes and the hybrid zone [33]. This suggests that clines at all nine markers examined here have been maintained due to selection despite gene flow. In accordance with their positions on the chromosome, likelihood ratio tests (LRTs) described three groups of markers that differed significantly from each other in shape and position (M1–M2, M3–M5, and D1–D4) (Figure 7). Maximum likelihood estimates of cline width revealed significantly steeper clines for M3–M5 (0.9–7.5 km) and D1–D4 (3.9–4.8 km) compared to M1–M2 (13.8–15.2 km) (Table S6), suggesting that similar patterns of intense selection contribute to the observed genetic variation at M3–M5 and D1–D4. Moreover, SNPs M3–M5 exhibit nearly complete fixation of alternate alleles at the tails of the distribution, which is consistent with our ecological expectations of divergent selection in alternate habitats due to different pollinators. Alternatively, even though D1–D4 are almost completely fixed for the *Y*-allele in eastern habitats, these markers display extensive variation in the western part of the range (Figure 8).

When the phenotypic cline in flower color estimated from these same populations was super-imposed on the allele frequency clines at these nine markers, the cline shapes for M1–M2 and D1–D4 were highly discordant with flower color (Figure 8). In particular, the centers of the M1–M2 and D1–D4 clines were shifted significantly to the east relative to flower color (Table S6). Moreover, LRTs revealed that only the clines at M3–M5 were not significantly different in shape and position compared to the phenotypic cline in flower color (Figure 7), resulting in nearly identical patterns of clinal variation between the phenotype and these genetic markers (Figure 8).

## Discussion

### A *cis*-regulatory change in *MaMyb2* confers pre-mating isolation during incipient speciation

While mutations in several genes are capable of generating the observed change in floral pigmentation between the ecotypes [38], our previous work in this system has shown that flower color differences are caused primarily by mutations in two genes likely encoding transcription factors that are responsible for differential regulation of the anthocyanin pathway enzymes in flowers [35]. At least one of these genes appears to be linked to *MaDfr*. Therefore, two lines of evidence were responsible for our initial hypothesis that *MaMyb2* was the major locus contributing to flower color differences between *M. aurantiacus* ecotypes: 1) sequence homology between *MaMyb2* and anthocyanin-regulating R2R3-MYB genes from other species (Figure 3), and 2) *MaMyb2* and *MaDfr* are genetically linked (Table S1). Three additional results obtained in this study provide consistent support for this hypothesis: 3) *MaMyb2* is integral to the regulatory network controlling floral anthocyanins; 4) *MaMyb2* regulates expression of *MaF3h, MaDfr*, and *MaAns*, which significantly alters floral pigmentation; and 5) a *cis*-acting mutation in *MaMyb2* is responsible for differences in *MaMyb2* gene expression.

Data obtained from genotype-phenotype associations from both a genetic cross in the laboratory and natural genetic variation in admixed hybrid zones consistently implicate *MaMyb2* in this phenotypic transition. Additionally, gene knock-down experiments provide compelling evidence that *MaMyb2* is necessary for the production of red flowers and is thus integral to the regulatory network controlling floral anthocyanins. Specifically, in both VIGS-silenced flowers (Figure 6B) as well as segregating F_3_ lines (Figure 5B), *MaMyb2* appears to regulate the expression of at least three enzymes (*MaF3h, MaDfr*, and *MaAns*) essential for anthocyanin biosynthesis. Furthermore, by demonstrating that *MaMyb2* expression differences between the ecotypes are due to a mutation (or mutations) that act in *cis* and not *trans*, we have established that genetic variation in *MaMyb2* itself and not a linked *trans*-factor is responsible for flower color differences. By contrast, both alleles at *MaDfr* are expressed at equivalent levels in F_1_ flowers [35], further suggesting that *MaDfr* is a regulatory target of *MaMyb2*. Finally, we have essentially ruled out coding mutations in *MaMyb2* as functionally related to the phenotype, providing further support that changes in *cis* to *MaMyb2* are responsible for ecotypic differences in flower color. While we have not yet identified the causal mutation (or mutations) responsible for this *cis*-acting expression change, it likely resides in a promoter or enhancer element that may be difficult to identify. Moreover, the remarkably tight genotype-phenotype associations in the hybrid zone at SNPs M3–M5 suggest that they are in linkage disequilibrium with the causal mutation. Therefore, functional tests in transgenic flowers will be necessary to determine which mutations are capable of activating *MaMyb2* expression.

It is worth noting that our initial results suggested that *MaDfr* could be responsible for the major flower color change [35]. While mutations of small effect in *MaDfr* acting additively or epistatically with *MaMyb2* cannot be ruled out from our current data, we show here that the major mutation lies within the linked *MaMyb2* gene and not *MaDfr*. Our data thus serve as a cautionary note of the potential to be misled by candidate genes and highlight the utility and necessity of collecting multiple pieces of corroborating genetic evidence to characterize the molecular basis of this trait. In doing so, we provide consistent support for a genetic model that explains the evolution of pre-mating isolation between two ecotypes at the early stages of divergence: a *cis*-regulatory mutation in *MaMyb2* is responsible for ecotypic differences in *MaMyb2* expression, which subsequently affects the regulation of *MaF3h, MaDfr*, and *MaAns* in flowers and leads to concomitant changes in floral anthocyanin pigmentation between the ecotypes.

### Divergent selection contributes to incipient species differences

Because *MaMyb2* contributes to adaptive flower color differences that appear to act as a partial barrier to gene flow between ecotypes, genetic variation in this locus is expected to exhibit a pattern of intense, spatially-structured differentiation across the geographic range of both ecotypes [21], [50], [51]. By taking complementary molecular population genetic approaches to test this hypothesis, our results provide support for this scenario and suggest that divergent selection on flower color has shaped the geographic distribution of *MaMyb2* alleles during these early stages of incipient species formation.

Theory suggests that population divergence can occur despite gene flow when divergent selection is strong. This is expected to result in a heterogeneous pattern of genomic differentiation, such that genetic variation at selected loci contributing to reproductive isolation will become highly differentiated, but genomic regions not affecting isolation will be permeable to gene flow (e.g. [52]). Although estimates of F_ST_ between the ecotypes are low at presumed neutral loci [33], we identified substantially elevated F_ST_ at all five genetic markers in *MaMyb2* (M1–M5) (Table 1). Thus, despite historically high levels of gene flow across the hybrid zone, ongoing selection appears to maintain genetic differentiation at *MaMyb2*. Surprisingly, although our genetic experiments have refuted *MaDfr*'s major involvement in flower color change, we also found very high allelic differentiation at D1–D4 (Table 1).

While these locus-specific patterns of elevated F_ST_ are indicative of positive selection, we further dissected the patterns of differential selection affecting the ecotypes by estimating the shape and geographic position of the allele frequency clines at these markers [47], [48]. Cline shape and position are directly related to the interaction between selection, which acts to maintain and steepen clines, and gene flow, which acts to widen or eliminate them [19], [20]. Thus, variation in the form and intensity of selection is expected to generate heterogeneity in introgression among loci, resulting in different cline shapes [49], [51]. For example, genetic incompatibilities that render hybrids unfit typically result in coincident clines across the majority of loci [50]. On the other hand, differences in the environment may favor alternate alleles at a locus, resulting in clines that vary in position and width according to the location and scale of the underlying environmental gradients [17]. Consequently, when intrinsic crossing barriers are absent, comparison of cline shapes and positions can describe the form and intensity of selection among loci.

The allele frequency clines among the nine SNPs group into three categories that differ significantly in shape, geographic position, and patterns of introgression (Figure 7), and these differences provide insight into the evolutionary processes that have generated these patterns.

For example, M1 and M2 are nearly fixed for *R* alleles in the west, but both *R* and *Y* alleles segregate in eastern populations (Figure 8). Indeed, among 267 red ecotype plants sampled from 16 populations, no *YY* homozygotes were found, resulting in a *R* allele frequency >0.996 (Table 1). By contrast, in 107 yellow ecotype individuals sampled from six populations, markers M1 and M2 segregated for all three genotypes, with the *R* allele frequency ranging between 0.158 (M1) and 0.256 (M2) (Table 1). Even though M1 and M2 are located within the *MaMyb2* gene, these patterns of genetic variation result in significantly wider clines that are east-shifted relative to the flower color cline (Figure 8), suggesting that these markers may have experienced a different evolutionary history that does not completely track the geographic variation in flower color.

Despite this apparent eastward introgression of *R* alleles at M1 and M2, we see the opposite pattern at D1–D4 (Table 1). Specifically, in the red ecotype populations, substantial genetic variation exists, with frequencies of the *R* allele ranging between 0.764 (D4) and 0.806 (D1) (Table 1). However, in the yellow ecotype populations, the *R* allele is nearly absent (<0.051) at all four markers (Table 1), with the *Y* allele achieving complete fixation in the most eastern populations (Figure 8). These patterns suggest asymmetries in the direction that alleles introgress at different loci. Moreover, they demonstrate that the opportunity for bi-directional gene flow exists across the hybrid zone. Nevertheless, *R* and *Y* alleles at M3–M5 are fixed or nearly fixed in their respective habitats, suggesting that introgression of non-native alleles at these loci is prevented due to selection. For markers M3–M5 only a single *RR* homozygote was found in yellow ecotype populations and no *YY* homozygotes were found in the red ecotype populations (Table 1), leading to exceptionally high allelic differentiation (e.g. F_ST_ at M5 is 1). We also detected tight concordance between the clines at M3–M5 and the phenotypic cline in flower color (Figure 7). This is consistent with the combined effect of strong divergent selection exerted by pollinators in alternate environments, an additive mode of gene action at *MaMyb2* (Figure 4A), and a series of three genetic markers in *MaMyb2* each in extremely tight linkage disequilibrium with the causal mutation. As a consequence, the resulting allele frequencies of markers M3–M5 almost perfectly track the geographic shift in flower color, reflecting the impact of divergent natural selection on this gene.

Variation in the shape and geographic position of clines among loci in *M. aurantiacus* contrasts with examples of clinal variation across other well-known hybrid zones. For example, in the classic house mouse hybrid zone between *Mus domesticus* and *M. musculus* across Europe, reproductive isolation arises mostly from intrinsic post-zygotic isolation due to genetic incompatibilities that evolved while in allopatry [53]. As a consequence of recent secondary contact and a balance between dispersal and hybrid unfitness, clines at most loci are concordant in shape and geographic position. In contrast, previous data on the *Mimulus aurantiacus* hybrid zone do not support a role for recent secondary contact following a long period of allopatry [33]. Instead, the hybrid zone appears to be maintained in the face of gene flow due primarily to divergent selection on traits such as flower color that confer strong, but incomplete pre-mating barriers. Therefore, if environmental heterogeneity exists across the hybrid zone, loci are likely to respond independently to multiple gradients of selection. This would explain the discord in patterns of introgression and cline center that we observe between markers M3–M5 and D1–D4 despite both sets of clines being very steep.

The recombination distance separating *MaMyb2* and *MaDfr* (11 cM) probably translates to a physical distance of several hundred kb or more. While the potential exists that epistasis and linkage lead to complex patterns of genetic variation across this region, it seems unlikely that the observed signatures of selection at D1–D4 are driven by hitch-hiking with *MaMyb2*. More likely, a single chromosomal region containing two candidate anthocyanin pathway genes appears to harbor independently selected loci, only one of which is directly related to incipient species differences in flower color. Future efforts will attempt to characterize the molecular basis of the interaction between *MaMyb2* and other loci that control flower color [35], as well as to characterize the genetic basis of other floral traits potentially involved in pollinator isolation. These and other population genomic analyses may help to ascertain the precise genetic target of selection being tracked by SNPs D1–D4 in order to determine its role in maintaining ecotypic differences.

### Conclusions

In this study, we genetically characterized an ecologically important transition in flower color between *M. aurantiacus* ecotypes that confers pre-mating isolation due to differences in pollinator preference. The combination of genetic mapping in the lab and field, differential gene expression, gene knockdowns, and allelic imbalance demonstrates consistent and compelling support that genetic variation in the form of a *cis*-acting mutation in *MaMyb2* is responsible for the major genetic transition in flower color between the red and yellow ecotypes. Our results also support the remarkable pattern of genetic convergence of flower color transitions identified previously among species [38]. Even though mutations in multiple genes can cause pigmentation differences, there is a significant fixation bias favoring mutations in R2R3-MYB genes, such that all documented transitions between species involve allelic differences in this family of transcription factor. Our results demonstrate that *MaMyb2* alleles have fixed differentially between ecotypes, providing further support for the prediction that natural selection has preferentially targeted these mutations because they have fewer deleterious pleiotropic effects relative to other mutations that cause similar changes in flower color [38], [40].

In addition, we used molecular population genetics to show that spatial variation in flower color across a natural hybrid zone was accompanied by concordant spatial variation of alleles in *MaMyb2*, reflecting a history of divergent selection on flower color. Our focus on a trait that diverged due to differential selection between two partially isolated ecotypes has provided us with a powerful opportunity to demonstrate the ecological and functional mechanisms controlling the evolution of reproductive isolation at the early stages of incipient speciation between these ecotypes. Ecologically based divergent selection is a pervasive feature of speciation [5]–[7], and the functional role of *MaMyb2* in pollinator isolation, coupled with the pattern of strong divergent selection indicated here, provides a rare example of a ‘speciation gene’ affecting pre-mating isolation during incipient species formation.

## Materials and Methods

### Identification of R2R3-MYB genes

Our previous work in *M. aurantiacus* suggested that a transcription factor linked to *MaDfr* was the major contributor of ecotypic differences in flower color. Therefore, we screened the *M. guttatus* genome (v. 1.1) to search for candidate anthocyanin genes linked to *MgDfr*. *MgDfr* occurs on linkage group 8 in *M. guttatus* [54] and is found on the third largest sequence scaffold (4.38 Mbp). No candidate anthocyanin genes were located on this scaffold. However, three floral anthocyanin regulators in the R2R3-MYB family of transcription factors (*MgMyb1-3*) were also shown to map to linkage group 8 but scaffold 11 (2.97 Mbp) [31]. Based on the known physical location of mapped markers on scaffold 11 in *M. guttatus* and the genetic map presented in [54], we deduced that *MgDfr* was approximately 101 cM from *MgMyb1-3*.

To identify the homologous R2R3-MYB genes in *M. aurantiacus*, we used 3′ Rapid Amplification of Complementary DNA (cDNA) Ends (3′ RACE) (Invitrogen). Total RNA was isolated from flower buds using the Spectrum Plant Total RNA kit (Sigma), followed by on-column DNase digestion. First-strand cDNA was synthesized using the Promega M-MLV reverse transcriptase. Two nested degenerate PCR primers were designed based on conserved regions of *MgMyb1-3*. PCR products were ligated into the pGEM-T-Easy vector (Promega) and inserts were sequenced from 14 plasmids. PCR primers for these and all subsequent experiments can be found in Table S7.

### Genetic mapping

*MaMyb1* was shown previously to be unlinked to *MaDfr* [35]. We genetically mapped *MaMyb2, MaMyb3* and *MaDfr* in 384 F_2_ hybrids. The details of the cross, development of the *MaDfr* genetic marker, DNA isolation, and flower color phenotyping (i.e. anthocyanin content) all have been described previously [35]. We identified SNPs from *MaMyb2* and *MaMyb3* that varied in the parents of this cross. Allelic differences were assayed using restriction digestion of PCR products followed by size separation with agarose gel electrophoresis. We tested the null hypothesis that segregation of each gene occurred independently.

### MaMyb2 sequencing

To obtain the complete coding sequence of *MaMyb2*, we performed 5′ RACE (Invitrogen) from floral cDNA. We then sequenced the entire gene from genomic DNA (gDNA) using species-specific primers from individuals from three different red and yellow ecotype populations (Table S4). In addition, we sequenced two individuals from different *M. aridus* populations (32.6538°, −116.1001°; and 32.6526°, −116.2449°) and one individual from *M. clevelandii* (33.1589°, −116.8122°) using the same primers. Gene trees of R2R3-MYB-related genes were constructed as described previously [35].

### Gene expression

We harvested flower buds from a red ecotype plant at 16 developmental stages. Total RNA was isolated and cDNA synthesized from eight stages from 250 ng RNA (Figure S3). PCR was carried out with varying cycle numbers using iProof High-Fidelity DNA polymerase (Bio-Rad) with primers designed using Primer Express v. 3.0 (Applied Biosystems). *Ef1a* controlled for cDNA quality, as described previously [35]. We qualitatively assayed for differences in *MaMyb2* floral expression using plants grown from seed in the University of Oregon greenhouses. Two flower buds per plant were harvested at stage 14 for RNA isolation and cDNA synthesis. PCR was carried out as described above. Strong and consistent differences in expression between the ecotypes at *MaF3h, MaDfr*, and *MaAns* were demonstrated previously [35], and were not replicated here. We performed quantitative real time PCR (QPCR) in 18 F_3_ hybrids of known genotype at *MaMyb2*, as described previously [35].

### Virus-induced gene silencing (VIGS)

We amplified a 324-bp fragment of *MaMyb2* using iProof High-Fidelity DNA polymerase from the full-length cDNA clone. The fragment was subcloned into the *BamHI*/*XhoI* sites of the pTRV2 vector (as described [43]) to generate the pTRV2-*MaMyb2* silencing construct. This construct shared little sequence similarity with *MaMyb1* and *MaMyb3* (Figure S1), indicating that, among the floral-expressed R2R3-MYBs, this silencing construct is likely specific for *MaMyb2*. The pTRV2-*MaMyb2*, pTRV2 (without the silencing construct) and pTRV1 (necessary for viral replication) vectors were transformed into *Agrobacterium tumefasciens* (strain GV3101). Single colonies containing each construct were used to inoculate 5 ml LB media plus antibiotics with shaking at 30°C. 4 ml pTRV1 and 3 ml pTRV2 and pTRV2-*MaMyb2* cultures were used to inoculate 500 ml LB+ antibiotics +10 mM MES +20 µM acetosyringone until OD_600_ = 1.0–1.2. Cells were harvested by centrifugation and pellets resuspended in infiltration solution (10 mM MES, 200 µM acetosyringone, 10 mM MgCl_2_,) to an OD_600_ = 2. Cells were incubated at room temperature for 4 hours before vacuum infiltration.

Seeds from the red ecotype were germinated on moist potting soil under artificial light in plug trays. At the 2–4 leaf stage, 384 seedlings were submerged in infiltration medium containing pTRV1 and pTRV2-*MaMyb2* mixed in a 1∶1 ratio with 0.005% Silwet L-70 and infiltrated under vacuum (20 in-Hg) for 3 min. As a negative control, 96 seedlings were treated with pTRV1 and pTRV2. Seedlings were re-potted and placed in a growth room at 25°C and sub-irrigated as necessary.

We collected entire corollas at stage 14 from nine plants treated with pTRV2-*MaMyb2* and four from pTRV2-treated negative control plants. As additional controls, corollas from four treated plants not showing altered flower colors and four plants not treated or vacuum infiltrated were collected. Total RNA was isolated and DNase-treated as described above. Validation of viral gene expression was detected using PCR from cDNA with primers specific for pTRV1 and pTRV2 plasmids (Figure S1). We used QPCR as described above to compare expression of *MaMyb2, MaF3h, MaDfr*, and *MaAns* between pTRV2-*MaMyb2* (N = 8) and pTRV2 (N = 4) treatments.

### Allelic imbalance

To compare expression of individual alleles, we determined the relative frequency that the *R* and *Y MaMyb2* alleles were cloned from PCR products of F_1_ cDNA and gDNA. Five F_1_ heterozygotes were generated from independent crosses between red and yellow ecotype individuals from separate populations. A single F_1_ from each cross was grown to flowering, and two flower buds were collected from each plant for RNA isolation and cDNA synthesis. In addition, gDNA was isolated from leaf tissue from each plant. We then used PCR to amplify a 198-bp region of the *MaMyb2* 5′ untranslated region containing a SNP that introduces an *HhaI* restriction site (marker M1; see Table S3 for details). PCR products were ligated into the pGem T-easy vector (Promega) and transformed into chemically competent *E. coli*. We performed a PCR using M13F and M13R vector-specific primers to amplify alleles from transformed colonies, followed by *HhaI* restriction digestion of the PCR product and agarose gel electrophoresis to count the relative frequency of *R* and *Y* alleles obtained from cDNA and gDNA. The following number of colonies were genotyped for each family (gDNA/cDNA): Family A, 80/70; Family B, 45/92; Family C, 45/92; Family D, 86/71; Family E, 37/22. We then used Fisher's exact test in each family to determine whether allele counts in gDNA and cDNA differed from each other and from equal representation.

### Natural population genotyping

Markers M1–M5 were identified from *MaMyb2* gDNA sequences. D1–D4 were designed by screening the annotated *M. guttatus* genome sequence (v. 1.1) on Phytozome v. 8.0 (www.phytozome.net). The *Dfr* gene in *M. guttatus* maps to scaffold 3, position: 4,218,020–4,220,275. We scanned 10 kb upstream and 10 kb downstream on scaffold 3 and identified four genes (including *MaDfr*) suitable for marker development (D1–D4). D1–D4 span four genes and 15-kb in *M. guttatus* and were identified from sequencing gDNA of homologous regions from *M. aurantiacus*. PCR primers and genotyping protocols for each marker are listed in Table S3. We confirmed that markers D1–D4 were linked to each other (as assumed based on their physical location in *M. guttatus*) by genotyping a panel of 24 F_2_s at markers D1–D4. No recombinants with *MaDfr* were identified. Genotypes from naturally occurring individuals were classified as *RR*, *RY*, or *YY* depending on whether alleles were derived from the red-ecotype (*R-allele*) or the yellow-ecotype (*Y-allele*) parents in these F_2_s.

We collected leaf tissue from 542 *M. aurantiacus* plants from 30 populations of both ecotypes and their hybrids (Table S4). Within the hybrid zone, we scored plants as *red, red-orange, orange*, or *yellow*. To confirm that these categories accurately reflected differences in anthocyanin content, we scored flowers from 66 plants from two hybrid populations (BS and WM) using the four categories and by quantitatively assaying anthocyanin content of each flower as described [35]. A one-way ANOVA was used to demonstrate that category significantly predicted quantitative anthocyanin content in each population (F*_BS_*_(3,61)_ = 105.6, *P*<0.0001, *R^2^* = 0.84; F*_WM_*_(3,62)_ = 232.4, *P*<0.0001, *R^2^* = 0.92;). F_ST_ between ecotypes was calculated for each of the nine markers from an analysis of molecular variance, as implemented in *GenoDive version 2.0b22* [55].

### Hybrid zone associations

Genotype-phenotype association studies were conducted in SAS using Fisher's exact test of flower color category vs. marker in the pooled hybrid dataset from the eight hybrid populations (Table S5).

### Estimation of cline shape parameters

We used an approach developed by Szymura and Barton [47] and modified by Porter et al. [48] to describe the relationship between allele frequency at the nine SNPs and geography. Cline shape is directly related to the interaction between selection, which acts to maintain and steepen clines, and gene flow, which acts to widen them. The model we used relates allele frequency (*p*) and geographic distance (*x*) by three equations: 
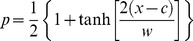
(1)

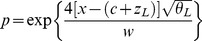
(2)

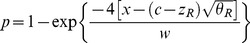
(3)where *c* describes the geographic position at which the maximum allele frequency gradient is observed (cline center), *w* (cline width) describes the geographic distance over which the maximum allele frequency change occurs (*w* = 1/slope at *c*), *θ_L_* and *θ_R_* describe the rate of exponential allele frequency decay on the left and right sides of the cline, respectively, and *z_L_* and *z_R_* describe the distance from *c* to a vertical asymptote for the exponential decay on the left and right sides of the cline, respectively.

These equations provide information on the shape, position, and patterns of introgression across the cline. Equation 1 provides the characteristic sigmoidal shape in the center of the cline, and equations 2 and 3 describe the exponential change in allele frequency on either side of the cline. The equations are related by the fact that as *θ* and *z* approach 1 and 0, respectively, equations 2 and 3 approach the shape of equation 1. Allele frequency was estimated for each SNP in each of the 30 populations. Because populations were sampled in two dimensions (north/south and east/west), but the major shift in flower color occurs in one dimension (east/west), population localities were assigned geographic distances along the east/west gradient based on the shortest, straight line distance of each population to the coast. Allele frequencies and geographic distance for each population and SNP are listed in Table S8. Distance metrics based on longitude provide qualitatively similar results. The six parameters described in the above equations were estimated using maximum likelihood (ML), as implemented in the program *ClineFit v. 0.2* (http://people.umass.edu/aporter/software/index.html) and described by Porter et al. [48].

For each of the nine SNPs, we used an eight parameter model with default settings to jointly estimate the above parameters along with the asymptotic allele frequencies on the left and right sides of the cline (*p_L_* and *p_R_*). For all SNPs except M1, the eight parameter model fit the data significantly better than a two parameter model that only estimates *c* and *w* (likelihood ratio tests of the full (eight-parameter) vs. reduced (two-parameter) model; M2–M5, D1–D4: *P*<0.05; M1: *P* = 0.053; 6 d.f.). Additional runs using either different random seeds or a greater number of sampling iterations provided similar outcomes. In addition to the nine SNP markers, we also obtained parameter estimates for the flower color cline from these same populations. Individuals were coded as one of three genotypes based on flower color: 1) red (*RR*); 2) red-orange and orange (*RY*); and 3) yellow (*YY*). Maximum likelihood estimates and 2-log-likelihood support values (analogous to 95% confidence intervals) for all parameters are presented in Table S6.

We then compared cline shapes among each SNP and the flower color cline using likelihood ratio tests (LRT). Because we estimated eight parameters, but only six parameters are necessary to define cline shape, we first fixed *p_L_* and *p_R_* to the ML estimates obtained from SNP *i* and then re-estimated the likelihood of observing the data at SNP *i* by constraining the six shape parameters to the ML estimates from SNP *j*. Thus, only the six shape parameters differed between the two models. Two times the differences in log-likelihood values for the freely estimated vs. constrained model were compared to a χ^2^ distribution with six degrees of freedom. LRTs were performed for all pairwise comparisons among the ten markers (nine SNPs and the flower color data), and statistical significance was determined after controlling for multiple comparisons using a Bonferroni-corrected α = 0.00056.

## Supporting Information

Figure S1 Methods for validation of VIGS silencing in *M. aurantiacus* flowers. (*A*) Representative pictures of *MaMyb2*-VIGS silencing in red ecotype plants. Top left picture shows the wild-type flower, and all other images demonstrate the range of silencing due to VIGS. (*B*) Validation of tobacco rattle virus (TRV) infection in the flowers among VIGS-treated plants. RNA was isolated from flower buds from four different treatments, as indicated, and described in the Materials and Methods. (+) represents plasmid DNA positive control for each of the three constructs (pTRV1 (top), pTRV2-negative control (bottom, left), and pTRV2-*MaMyb2* (bottom, right)). Top lanes are PCR using primers pTRV1 F/R to test for the presence of the pTRV1 genes. Bottom lanes are PCR using primers pTRV2 F/R to test for the presence of pTRV2 genes with or without the *MaMyb2* silencing construct. Presence of PCR products with both primer pairs indicates successful TRV infection and replicating virus in plant cells. All samples that were untreated were negative for both PCR products. All tested plants containing a *MaMyb2* silencing construct that were positive for both PCR products showed altered flower color phenotypes, demonstrating positive VIGS silencing. 2-log DNA ladder (New England Biolabs) is shown in the first lane of each row. (*C*) Partial DNA sequence alignment of the coding regions of *MaMyb1, MaMyb2*, and *MaMyb3*. Nucleotide position, in base-pairs, is indicated. Dots indicate sequence conservation with the *MaMyb2* sequence, and dashes indicate alignment gaps. The alignment was generated first from amino acid sequences and then translated back to nucleotide sequences. The location of the 324-bp *MaMyb2* VIGS silencing construct is highlighted in gray. Virtually no sequence conservation exists in this portion of the alignment among floral-expressed MYB genes in *M. aurantiacus*.
(EPS)

Figure S2 *(A)* Alignment of the predicted amino acid sequences from the red and yellow ecotypes at *MaMyb2*. Dots indicate sequence conservation with the red ecotype sequences and dashes indicate alignment gaps. The nucleotide sequences, including the introns, are published in Genbank. *(B)* Comparative sequence analysis of coding mutations in *MaMyb2*. Shown are the amino acid positions that vary between the red and yellow ecotype sequences for 3 red ecotype, 3 yellow ecotype, 2 *M. aridus*, and 1 *M. clevelandii* individuals. Letters represent the amino acid at that position and dashes indicate gaps due to insertion-deletion mutations. The color column represents the flower color of each taxon.
(EPS)

Figure S3 Time series of flower bud development and corresponding changes in anthocyanin gene expression. Flower buds were collected from a red ecotype plant at 16 developmental stages, beginning with the smallest visible bud (Stage 1). Anthocyanins first become visible in the developing bud at Stage 8. From eight of these stages, we examined qualitative expression levels from the genes indicated. Stage number is listed above each lane, and PCR cycle number is indicated beside each gene name. All genes are expressed at high levels through stage 15. We collected tissue for subsequent RNA isolation and expression analyses at stage 14.
(EPS)

Table S1 Genetic linkage analysis of *MaDfr, MaMyb2*, and *MaMyb3*. We tested the null hypothesis that segregation of each gene occurred independently. Observed and expected genotypes are presented for each pairwise comparison of the three genes, with SNP nucleotide genotypes indicated.
(DOCX)

Table S2 Results of genetic analyses comparing genotype at *MaDfr, MaMyb2*, and *MaMyb3* with flower color in F_2_ hybrids. Flower color is measured as the relative anthocyanin content extracted from flowers.
(DOCX)

Table S3 Details of the nine SNP markers used for hybrid zone genotype-phenotype association studies and cline shape analyses. A) The five SNPs from *MaMyb2*. The nucleotide position, location, PCR genotyping primers, and restriction enzymes used for genotyping are indicated. B) The four markers surrounding *MaDfr*. The *M. guttatus* scaffold 3 position of the homologous gene containing the SNP, the *M. guttatus* transcript name, annotation, *M. aurantiacus* PCR primers and genotyping conditions are indicated.
(DOCX)

Table S4 Collection information for the 30 populations used for hybrid zone genotype-phenotype association studies and cline shape analyses. R = red ecotype, Y = yellow ecotype, H = hybrid population. Sample size is the number of individuals used in the final genotype data set.
(DOCX)

Table S5 Flower color counts for each genotype and SNP marker combination from hybrid zone genotype-phenotype association studies. Statistical significance among genotypes was tested using Fisher's exact test.
(DOCX)

Table S6 Maximum likelihood estimates of cline shape parameters for each of the nine SNP markers and the flower color (FC) data. 2-log-likelihood support limits are in parentheses. Parameter abbreviations and descriptions can be found in the Methods.
(DOCX)

Table S7 PCR primer sequences used in this study.
(DOCX)

Table S8 Geographic distance from the coast and *R-allele* frequency for the nine SNPs in each of 30 populations. Location and other collection information are presented in Table S4.
(DOCX)
