# Supplementary material for: Divergent Selection Drives Genetic Differentiation in an R2R3-MYB Transcription Factor That Contributes to Incipient Speciation in Mimulus aurantiacus
Source: PLoS Genet. 2013 Mar 21;9(3):e1003385. doi: 10.1371/journal.pgen.1003385 (PMC3605050; doi:10.1371/journal.pgen.1003385)
Supplement: Table S1 — Genetic linkage analysis of MaDfr, MaMyb2, and MaMyb3. We tested the null hypothesis that segregation of each gene occurred independently. Observed and expected genotypes are presented for each pairwise comparison of the three genes, with SNP nucleotide genotypes indicated. (DOCX) [file pgen.1003385.s004.docx]

| ***MaDfr*** | ***MaMyb2*** | **Observed** | | **Expected** | | | **X^2^** | | ***P* (8 d.f.)** |
| --- | --- | --- | --- | --- | --- | --- | --- | --- | --- |
| **TT** | AA | 68 | | 22.5 | | | 301.64 | | 1.8 x 10^-60^ |
| **TT** | AG | 10 | | 45 | | |  | |  |
| **TT** | GG | 1 | | 22.5 | | |  | |  |
| **TC** | AA | 22 | | 45 | | |  | |  |
| **TC** | AG | 152 | | 90 | | |  | |  |
| **TC** | GG | 25 | | 45 | | |  | |  |
| **CC** | AA | 4 | | 22.5 | | |  | |  |
| **CC** | AG | 17 | | 45 | | |  | |  |
| **CC** | GG | 61 | | 22.5 | | |  | |  |
|  |  |  | | | | | |  | |
| ***MaDfr*** | ***MaMyb3*** | **Observed** | | | **Expected** | **X^2^** | | | ***P* (8 d.f.)** |
| **TT** | TT | 21 | | | 21.88 | 12.70 | | | 0.122 |
| **TT** | AT | 42 | | | 43.75 |  | | |  |
| **TT** | AA | 16 | | | 21.88 |  | | |  |
| **TC** | TT | 53 | | | 43.75 |  | | |  |
| **TC** | AT | 82 | | | 87.5 |  | | |  |
| **TC** | AA | 60 | | | 43.75 |  | | |  |
| **CC** | TT | 15 | | | 21.88 |  | | |  |
| **CC** | AT | 39 | | | 43.75 |  | | |  |
| **CC** | AA | 22 | | | 21.88 |  | | |  |
|  |  | |  | | | | |  | |
| ***MaMyb2*** | ***MaMyb3*** | **Observed** | | | **Expected** | | **X^2^** | | ***P* (8 d.f.)** |
| **AA** | TT | 28 | | | 20.5 | | 8.30 | | 0.404 |
| **AG** | AT | 42 | | | 41 | |  | |  |
| **GG** | AA | 18 | | | 20.5 | |  | |  |
| **AA** | TT | 42 | | | 41 | |  | |  |
| **AG** | AT | 71 | | | 82 | |  | |  |
| **GG** | AA | 49 | | | 41 | |  | |  |
| **AA** | TT | 15 | | | 20.5 | |  | |  |
| **AG** | AT | 39 | | | 41 | |  | |  |
| **GG** | AA | 24 | | | 20.5 | |  | |  |
