# Supplementary material for: Divergent Selection Drives Genetic Differentiation in an R2R3-MYB Transcription Factor That Contributes to Incipient Speciation in Mimulus aurantiacus
Source: PLoS Genet. 2013 Mar 21;9(3):e1003385. doi: 10.1371/journal.pgen.1003385 (PMC3605050; doi:10.1371/journal.pgen.1003385)
Supplement: Table S2 — Results of genetic analyses comparing genotype at MaDfr, MaMyb2, and MaMyb3 with flower color in F2 hybrids. Flower color is measured as the relative anthocyanin content extracted from flowers. (DOCX) [file pgen.1003385.s005.docx]

| ***MaDfr*** | **N** | **Mean (S.D.)** | **F_(2, 381)_** | ***P*** |
| --- | --- | --- | --- | --- |
| **TT** | 86 | 0.385 (0.244) | 126.68 | <0.0001 |
| **CT** | 213 | 0.119 (0.128) |  |  |
| **CC** | 85 | 0.035 (0.078) |  |  |
|  |  |  |  |  |
| ***MaMyb2*** | **N** | **Mean (S.D.)** | **F_(2, 357)_** | ***P*** |
| **AA** | 94 | 0.385 (0.245) | 176.33 | <0.0001 |
| **AG** | 179 | 0.115 (0.085) |  |  |
| **GG** | 87 | 0.016 (0.053) |  |  |
|  |  |  |  |  |
| ***MaMyb3*** | **N** | **Mean (S.D.)** | **F_(2, 347)_** | ***P*** |
| **TT** | 89 | 0.163 (0.183) | 0.811 | 0.445 |
| **AT** | 163 | 0.173 (0.225) |  |  |
| **AA** | 98 | 0.140 (0.179) |  |  |
