# Supplementary material for: Divergent Selection Drives Genetic Differentiation in an R2R3-MYB Transcription Factor That Contributes to Incipient Speciation in Mimulus aurantiacus
Source: PLoS Genet. 2013 Mar 21;9(3):e1003385. doi: 10.1371/journal.pgen.1003385 (PMC3605050; doi:10.1371/journal.pgen.1003385)
Supplement: Table S3 — Details of the nine SNP markers used for hybrid zone genotype-phenotype association studies and cline shape analyses. A) The five SNPs from MaMyb2. The nucleotide position, location, PCR genotyping primers, and restriction enzymes used for genotyping are indicated. B) The four markers surrounding MaDfr. The M. guttatus scaffold 3 position of the homologous gene containing the SNP, the M. guttatus transcript name, annotation, M. aurantiacus PCR primers and genotyping conditions are indicated. (DOCX) [file pgen.1003385.s006.docx]

A)

| **Marker** | **Position** | **Location** | **Genotyping Primers (5' – 3’)** | **Restriction**  **enzyme** |
| --- | --- | --- | --- | --- |
| **M1** | 120 | 5' UTR | CTACTCAAATGTCTTCGTCTTAGCCATTTGCG;  AGCTCTGAGAGGGACAAGAT | *HhaI* |
|  |  |  |  |  |
| **M2** | 226 | 1st exon | GTCGACAAATTTGGAGAAGGG;  CTCCAAATTTGTCGACGCATTTCCTCAGC | *PvuII* |
|  |  |  |  |  |
| **M3** | 746 | Second Intron | CGAAGCTGATCTCATTCTCAGGCTT;  CGGCAGTGGTGGCTGGTGACTT | *HhaI* |
|  |  |  |  |  |
| **M4** | 975 | Second Intron | TTGGGTACTGACCTAGTTGG CGGCAGTGGTGGCTGGTGACTT | *NsiI* |
|  |  |  |  |  |
| **M5** | 1502 | Third Exon | CATTCGTTGGTGGAGCAACTTGCT;  CTTTGGAGGAATAGTCCAAGT | *XbaI* |

B)

| **Marker** | ***M. guttatus***  **Scaffold 3 Position** | ***M. guttatus***  **Transcript** | **Annotation** | **Genotyping Primers**  **(5' – 3’)** | **Restriction enzyme** |
| --- | --- | --- | --- | --- | --- |
| **D1** | 4208660-4210968 | *mgv1a007062m* | GTP-binding protein | GATAAGCCTCAGACTACTAGAC;  GCATTTACGGCAGCACTTCGTAC | *AseI* |
|  |  |  |  |  |  |
| **D2** | 4213349-4216303 | *mgv1a006512m* | No functional annotation | GGATAGACTGATAGAACCTG;  GAGATAGTTGTTCGCGTATCCTCTG | *BclI* |
|  |  |  |  |  |  |
| **D3** | 4218020-4220275 | *mgv1a006799m* | dihydroflavonol 4-reductase (DFR) | TGCAACTTTGTTCGCTTAAA;  TCACCGTTCAGTGTTGGGTA | *ClaI* |
|  |  |  |  |  |  |
| **D4** | 4220577-4223597 | *mgv1a012119m* | Sugar 1-phoshpate guanyl transferase | TGTCCGACAACATACAAGAA;  GAGCCTGAAGTACAGGATTG | *in/del* |
