# Supplementary material for: Divergent Selection Drives Genetic Differentiation in an R2R3-MYB Transcription Factor That Contributes to Incipient Speciation in Mimulus aurantiacus
Source: PLoS Genet. 2013 Mar 21;9(3):e1003385. doi: 10.1371/journal.pgen.1003385 (PMC3605050; doi:10.1371/journal.pgen.1003385)
Supplement: Table S4 — Collection information for the 30 populations used for hybrid zone genotype-phenotype association studies and cline shape analyses. R = red ecotype, Y = yellow ecotype, H = hybrid population. Sample size is the number of individuals used in the final genotype data set. (DOCX) [file pgen.1003385.s007.docx]

| **Population** | **Latitude**  **(Degrees)** | **Longitude**  **(Degrees)** | **Ecotype** | **Sample Size** |
| --- | --- | --- | --- | --- |
| **BCG** | 32.63108 | -117.02170 | R | 16 |
| **CLC** | 32.92045 | -117.16942 | R | 21 |
| **CRS** | 33.13037 | -117.30717 | R | 15 |
| **DLR** | 33.16818 | -117.05237 | R | 16 |
| **EHP** | 32.72317 | -117.07298 | R | 8 |
| **ELF** | 33.08595 | -117.14530 | R | 24 |
| **ELT** | 32.89422 | -117.08982 | R | 16 |
| **FLP** | 32.80582 | -116.98670 | R | 16 |
| **LDG** | 32.72752 | -116.97810 | R | 16 |
| **LH** | 33.06088 | -117.11877 | R | 24 |
| **MT** | 32.82095 | -117.06175 | R | 16 |
| **OSP** | 33.10197 | -117.03508 | R | 8 |
| **PMD** | 32.93787 | -117.05913 | R | 16 |
| **SDP** | 32.99810 | -117.23538 | R | 16 |
| **SXN** | 33.07717 | -117.28523 | R | 7 |
| **UCSD** | 32.88940 | -117.23618 | R | 32 |
| **BC** | 33.12262 | -116.80468 | H | 24 |
| **BS** | 33.01480 | -117.01643 | H | 24 |
| **DLZ** | 32.65250 | -116.78597 | H | 9 |
| **JMC** | 32.73732 | -116.95410 | H | 24 |
| **LKW** | 33.16372 | -117.01610 | H | 24 |
| **MW** | 33.00718 | -116.95978 | H | 23 |
| **OAK** | 32.91407 | -116.88932 | H | 16 |
| **WM** | 32.82133 | -116.90228 | H | 24 |
| **BCRD** | 32.94958 | -116.63795 | Y | 8 |
| **INJ** | 33.09785 | -116.66432 | Y | 16 |
| **LO** | 32.67670 | -116.33123 | Y | 23 |
| **PCT** | 32.73258 | -116.46983 | Y | 21 |
| **POTR** | 32.60380 | -116.63392 | Y | 23 |
| **PVT** | 32.83340 | -116.54860 | Y | 16 |
