# Supplementary material for: Divergent Selection Drives Genetic Differentiation in an R2R3-MYB Transcription Factor That Contributes to Incipient Speciation in Mimulus aurantiacus
Source: PLoS Genet. 2013 Mar 21;9(3):e1003385. doi: 10.1371/journal.pgen.1003385 (PMC3605050; doi:10.1371/journal.pgen.1003385)
Supplement: Table S5 — Flower color counts for each genotype and SNP marker combination from hybrid zone genotype-phenotype association studies. Statistical significance among genotypes was tested using Fisher's exact test. (DOCX) [file pgen.1003385.s008.docx]

| **SNP** | **Genotype** | **Red**  **(N=50)** | **Red/Orange (N=27)** | **Orange (N=30)** | **Yellow (N= 61)** | ***P*** |
| --- | --- | --- | --- | --- | --- | --- |
| **M1** | RR | 49 | 26 | 28 | 32 | 1.9 x 10^-9^ |
|  | RY | 1 | 1 | 2 | 26 |  |
|  | YY | 0 | 0 | 0 | 3 |  |
|  |  |  |  |  |  |  |
| **M2** | RR | 50 | 27 | 30 | 41 | 3.2 x 10^-8^ |
|  | RY | 0 | 0 | 0 | 18 |  |
|  | YY | 0 | 0 | 0 | 2 |  |
|  |  |  |  |  |  |  |
| **M3** | RR | 48 | 17 | 2 | 0 | 1.9 x 10^-55^ |
|  | RY | 2 | 10 | 25 | 1 |  |
|  | YY | 0 | 0 | 3 | 59 |  |
|  |  |  |  |  |  |  |
| **M4** | RR | 48 | 18 | 3 | 5 | 5.1 x 10^-43^ |
|  | RY | 1 | 8 | 27 | 9 |  |
|  | YY | 0 | 0 | 0 | 46 |  |
|  |  |  |  |  |  |  |
| **M5** | RR | 48 | 18 | 7 | 0 | 6.7 x 10^-52^ |
|  | RY | 2 | 9 | 21 | 2 |  |
|  | YY | 0 | 0 | 0 | 58 |  |
|  |  |  |  |  |  |  |
| **D1** | RR | 30 | 16 | 7 | 9 | 4.1 x 10^-9^ |
|  | RY | 19 | 11 | 17 | 30 |  |
|  | YY | 1 | 0 | 6 | 22 |  |
|  |  |  |  |  |  |  |
| **D2** | RR | 30 | 16 | 7 | 10 | 1.0 x 10^-8^ |
|  | RY | 19 | 11 | 17 | 29 |  |
|  | YY | 1 | 0 | 6 | 22 |  |
|  |  |  |  |  |  |  |
| **D3** | RR | 26 | 17 | 10 | 18 | 3.8 x 10^-5^ |
|  | RY | 22 | 10 | 14 | 21 |  |
|  | YY | 1 | 0 | 6 | 21 |  |
|  |  |  |  |  |  |  |
| **D4** | RR | 29 | 15 | 7 | 10 | 1.8 x 10^-7^ |
|  | RY | 19 | 12 | 18 | 29 |  |
|  | YY | 2 | 0 | 5 | 21 |  |
