# Supplementary material for: Divergent Selection Drives Genetic Differentiation in an R2R3-MYB Transcription Factor That Contributes to Incipient Speciation in Mimulus aurantiacus
Source: PLoS Genet. 2013 Mar 21;9(3):e1003385. doi: 10.1371/journal.pgen.1003385 (PMC3605050; doi:10.1371/journal.pgen.1003385)
Supplement: Table S6 — Maximum likelihood estimates of cline shape parameters for each of the nine SNP markers and the flower color (FC) data. 2-log-likelihood support limits are in parentheses. Parameter abbreviations and descriptions can be found in the Methods. (DOCX) [file pgen.1003385.s009.docx]

| **Marker** | ***c*** | | ***w*** | ***θ_L_*** | ***θ_R_*** | ***z_L_*** | ***z_R_*** | ***p_L_*** | ***p_R_*** |
| --- | --- | --- | --- | --- | --- | --- | --- | --- | --- |
| **M1** | 38.95 | | 15.20 | 0.3517 | 0.0172 | 24.48 | 20.08 | 1.28x10^-5^ | 0.994 |
|  | (35.39, 42.24) | | (11.21, 18.76) | (0.0523, 0.7439) | (0.0080, 0.0373) | (4.58, 102.82) | (14.35, 32.01) | (6.33x10^-8^, 0.002) | (0.826, 0.999) |
| **M2** | 43.73 | | 13.84 | 0.0006 | 0.0180 | 990.37 | 17.87 | 0.0011 | 0.977 |
|  | (38.70, 46.17) | | (8.51, 18.59) | (0.0003, 0.7201) | (0.0067, 0.0548) | (5.00, 999.83) | (10.61, 33.06) | (2.70x10^-6^, 0.010) | (0.768, 0.999) |
| **M3** | 27.48 | | 3.91 | 0.1550 | 0.0204 | 3.37 | 5.90 | 5.16x10^-8^ | 0.943 |
|  | (26.94, 28.51) | | (2.85, 6.42) | (0.0823, 0.4989) | (0.0086, 0.0867) | (1.46, 6.19) | (3.31, 12.880) | (5.02x10^-8^, 0.001 | (0.896, 0.975) |
| **M4** | 28.92 | | 7.54 | 0.1446 | 0.0753 | 25.79 | 4.76 | 1.37x10^-7^ | 0.920 |
|  | (27.69, 30.14) | | (4.87, 10.39) | (0.0030, 0.7493) | (0.0270, 0.3915) | (2.23, 394.46) | (3.28, 17.64) | (1.370x10^-7^, 0.001) | (0.865, 0.960) |
| **M5** | 26.49 | | 0.89 | 0.0257 | 0.0006 | 3.13 | 6.47 | 0.0093 | 0.937 |
|  | (26.32, 28.66) | | (0.48, 5.29) | (0.0008, 0.9996) | (0.0001, 0.0311) | (1.04, 999.90) | (4.41, 12.30) | (0.006, 0.024) | (0.885, 0.975) |
| **FC** | 26.31 | | 0.81 | 0.0015 | 0.0008 | 10.33 | 4.94 | 1.51x10^-8^ | 1.000 |
|  | (26.19, 26.97) | | (0.58, 2.90) | (0.0008, 0.0287) | (0.0004, 0.0122) | (6.04, 16.27) | (3.94, 8.78) | (1.51x10^-8^, 0.01) | (0.983, 1.000) |
| **D1** | 46.05 | | 4.83 | 0.0010 | 0.5829 | 27.09 | 68.15 | 0.029 | 1.000 |
|  | (45.73, 49.23) | | (2.93, 7.78) | (0.0005, 0.0046) | (0.0073, 0.9966) | (19.38, 39.43) | (2.30, 675.64) | (0.001, 0.168) | (0.978, 1.000) |
| **D2** | 46.05 | | 4.76 | 0.0011 | 0.0275 | 24.36 | 98.78 | 0.054 | 1.000 |
|  | (45.69, 50.23) | | (2.71, 7.71) | (0.0006, 0.0044) | (0.0002, 0.9962) | (18.34, 34.88) | (2.24, 928.95) | (0.006, 0.173) | (0.979, 1.000) |
| **D3** | 46.06 | | 3.90 | 0.0006 | 0.3281 | 28.03 | 104.36 | 0.035 | 1.000 |
|  | (45.81, 49.02) | | (2.34, 7.48) | (0.0003, 0.0064) | (0.1113, 0.9290) | (16.97, 32.49) | (18.45, 107.58) | (7.95x10^-5,^ 0.184) | (0.977, 1.000) |
| **D4** | 46.05 | | 4.43 | 0.0006 | 0.4337 | 32.78 | 35.26 | 0.0291 | 1.000 |
|  | | (45.75, 51.19) | (2.30, 7.75) | (0.0003, 0.0035) | (0.0092, 0.9993) | (19.98, 44.50) | (1.53, 434.73) | (0.0002, 0.1938) | (0.978, 1.000) |
