# Supplementary material for: Divergent Selection Drives Genetic Differentiation in an R2R3-MYB Transcription Factor That Contributes to Incipient Speciation in Mimulus aurantiacus
Source: PLoS Genet. 2013 Mar 21;9(3):e1003385. doi: 10.1371/journal.pgen.1003385 (PMC3605050; doi:10.1371/journal.pgen.1003385)
Supplement: Table S7 — PCR primer sequences used in this study. (DOCX) [file pgen.1003385.s010.docx]

|  | **Primer sequence (5'-3')** |
| --- | --- |
| ***MaMyb2* cloning** |  |
| **Myb_deg-F1** | GGTGCATGGACAAAAGAMGAAG |
| **Myb_deg-F2** | AARTGGCATCTWGTCCCTCT |
| **5' RACE Myb2 GSP** | CGGCAGTGGTGGCTGGTGACTT |
|  |  |
| ***MaMyb2* gDNA sequencing** |  |
| **MaMyb2(1-293)-F** | GCATGCATATATGTTCCATCAA |
| **MaMyb2(1-293)-R** | CTCAGACCCAATCTCAAAAGAGGT |
| **MaMyb2(188-561)-F** | CATTTTAATTTGATATTTTGCATG |
| **MaMyb2(188-561)-R** | CCTAGTTGGTAGAGTAAGAATAGC |
| **MaMyb2(550-1553)-F** | TTGGGTACTGACCTAGTTGG |
| **MaMyb2(550-1553)-R** | ACTTGGACTATTCCTCCAAAG |
|  |  |
| ***MaMyb3* genotyping** |  |
| **MaMyb3-F** | CCTCCGCTGGATCAACTATC |
| **MaMyb3-R** | AGCTATAAGTGACCACCTAATTAATTTAAA |
|  |  |
| **VIGS cloning and validation** |  |
| **VIGS_BamHI** | ACGTAGGATCCTCCAAATCACAAGTACCGGTA |
| **VIGS_XhoI** | TGATGATCGTACAACCAACTCTCGAGTGCAT |
| **pTRV1-F** | GTTGGTGGGAAGAAGAGTGAAC |
| **pTRV1-R** | AGTGTCGCTAATATGGGTTGCT |
| **pTRV2-F** | GGTCAAGGTACGTAGTAGAG |
| **pTRV2-R** | CGAGAATGTCAATCTCGTAGG |
|  |  |
| **Gene expression** |  |
| **MaF3H-F** | AATGCCCTCAGCCCAATCT |
| **MaF3H-R** | TGATCCATGTCTTCCCACCAT |
| **MaDfr-F** | AGCAGCAATTGAAGCAGCTAAA |
| **MaDfr-R** | GTAATGGGTGAAAGTGCAGTGATC |
| **MaAns-F** | AAAAGACGATCTGATCCTCCAAAT |
| **MaAns-R** | ACCATGTTGTGGAGGATGAATG |
| **MaMyb2-F** | TCCGAAGACCGGTGAGCC |
| **MaMyb2-R** | TTTTCTTCCTCGTTTTCACTCA |
| **Ef1a-F** | AAATTCCGTTTGTCCCCATTT |
| **Ef1a-R** | GCCTCTTGGGCTCACTGATC |
