# Supplementary material for: Divergent Selection Drives Genetic Differentiation in an R2R3-MYB Transcription Factor That Contributes to Incipient Speciation in Mimulus aurantiacus
Source: PLoS Genet. 2013 Mar 21;9(3):e1003385. doi: 10.1371/journal.pgen.1003385 (PMC3605050; doi:10.1371/journal.pgen.1003385)
Supplement: Table S8 — Geographic distance from the coast and R-allele frequency for the nine SNPs in each of 30 populations. Location and other collection information are presented in Table S4. (DOCX) [file pgen.1003385.s011.docx]

| **Population** | **Ecotype** | **Distance**  **(km)** | **M1** | **M2** | **M3** | **M4** | **M5** | **D1** | **D2** | **D3** | **D4** |
| --- | --- | --- | --- | --- | --- | --- | --- | --- | --- | --- | --- |
| **BCG** | R | 7.25 | 1.00 | 1.00 | 1.00 | 1.00 | 1.00 | 0.81 | 0.80 | 0.81 | 0.81 |
| **CLC** | R | 8.15 | 1.00 | 1.00 | 1.00 | 1.00 | 1.00 | 0.81 | 0.83 | 0.83 | 0.81 |
| **CRS** | R | 2.31 | 0.97 | 0.97 | 0.93 | 1.00 | 1.00 | 0.83 | 0.82 | 0.83 | 0.83 |
| **DLR** | R | 25.92 | 1.00 | 1.00 | 1.00 | 1.00 | 1.00 | 0.90 | 0.91 | 0.91 | 0.91 |
| **EHP** | R | 7.02 | 1.00 | 1.00 | 1.00 | 1.00 | 1.00 | 0.56 | 0.56 | 0.44 | 0.75 |
| **ELF** | R | 14.89 | 0.98 | 1.00 | 0.98 | 1.00 | 1.00 | 0.69 | 0.69 | 0.71 | 0.71 |
| **ELT** | R | 15.29 | 1.00 | 1.00 | 1.00 | 1.00 | 1.00 | 0.97 | 0.97 | 0.97 | 0.97 |
| **FLP** | R | 19.25 | 1.00 | 1.00 | 1.00 | 1.00 | 1.00 | 1.00 | 1.00 | 1.00 | 1.00 |
| **LDG** | R | 14.63 | 1.00 | 1.00 | 1.00 | 1.00 | 1.00 | 0.69 | 0.69 | 0.69 | 0.63 |
| **LH** | R | 16.18 | 1.00 | 1.00 | 1.00 | 1.00 | 1.00 | 0.88 | 0.73 | 0.77 | 0.65 |
| **MT** | R | 14.27 | 1.00 | 1.00 | 1.00 | 1.00 | 1.00 | 0.72 | 0.72 | 0.59 | 0.59 |
| **OSP** | R | 25.43 | 1.00 | 1.00 | 1.00 | 1.00 | 1.00 | 0.64 | 0.64 | 0.63 | 0.63 |
| **PMD** | R | 19.10 | 1.00 | 1.00 | 1.00 | 1.00 | 1.00 | 0.75 | 0.75 | 0.66 | 0.59 |
| **SDP** | R | 3.45 | 1.00 | 1.00 | 1.00 | 1.00 | 1.00 | 0.81 | 0.75 | 0.81 | 0.78 |
| **SXN** | R | 2.08 | 1.00 | 1.00 | 1.00 | 1.00 | 1.00 | 0.79 | 0.79 | 0.93 | 0.93 |
| **UCSD** | R | 1.55 | 1.00 | 1.00 | 1.00 | 1.00 | 1.00 | 0.83 | 0.83 | 0.77 | 0.75 |
| **BC** | H | 46.05 | 0.63 | 0.63 | 0.04 | 0.06 | 0.04 | 0.77 | 0.77 | 0.73 | 0.75 |
| **BS** | H | 24.31 | 1.00 | 1.00 | 0.88 | 0.88 | 0.89 | 0.35 | 0.33 | 0.38 | 0.33 |
| **DLZ** | H | 29.62 | 0.56 | 0.94 | 0.28 | 0.29 | 0.28 | 0.50 | 0.56 | 0.63 | 0.56 |
| **JMC** | H | 17.04 | 1.00 | 1.00 | 0.96 | 1.00 | 0.96 | 0.79 | 0.79 | 0.76 | 0.77 |
| **LKW** | H | 28.93 | 0.98 | 1.00 | 0.22 | 0.48 | 0.29 | 0.81 | 0.81 | 0.83 | 0.79 |
| **MW** | H | 29.24 | 0.91 | 0.98 | 0.37 | 0.44 | 0.41 | 0.59 | 0.59 | 0.65 | 0.61 |
| **OAK** | H | 32.55 | 0.91 | 0.97 | 0.53 | 0.63 | 0.53 | 0.38 | 0.38 | 0.41 | 0.40 |
| **WM** | H | 26.17 | 0.96 | 0.98 | 0.69 | 0.71 | 0.77 | 0.46 | 0.48 | 0.58 | 0.48 |
| **BCRD** | Y | 54.71 | 0.00 | 0.06 | 0.00 | 0.00 | 0.00 | 0.00 | 0.00 | 0.00 | 0.00 |
| **INJ** | Y | 58.60 | 0.19 | 0.13 | 0.00 | 0.03 | 0.00 | 0.00 | 0.00 | 0.00 | 0.00 |
| **LO** | Y | 72.59 | 0.00 | 0.04 | 0.00 | 0.00 | 0.00 | 0.00 | 0.00 | 0.00 | 0.00 |
| **PCT** | Y | 60.41 | 0.39 | 0.62 | 0.00 | 0.05 | 0.00 | 0.00 | 0.00 | 0.00 | 0.00 |
| **POTR** | Y | 43.74 | 0.25 | 0.44 | 0.00 | 0.02 | 0.00 | 0.16 | 0.17 | 0.24 | 0.16 |
| **PVT** | Y | 56.61 | 0.07 | 0.25 | 0.04 | 0.06 | 0.00 | 0.00 | 0.00 | 0.00 | 0.00 |
